# The impacts of diel thermal variability on growth, development and performance of wild Atlantic salmon (*Salmo salar*) from two thermally distinct rivers

**DOI:** 10.1093/conphys/coae007

**Published:** 2024-02-12

**Authors:** Sean Andrew, Sula Swart, Stephanie McKenna, Jenna Morissette, Carole-Anne Gillis, Tommi Linnansaari, Suzanne Currie, Andrea J Morash

**Affiliations:** Department of Biology, Mount Allison University, 62 York St., Sackville, NB E4L 1G7, Canada; Department of Biology, Mount Allison University, 62 York St., Sackville, NB E4L 1G7, Canada; Department of Biology, Mount Allison University, 62 York St., Sackville, NB E4L 1G7, Canada; Department of Biology, Mount Allison University, 62 York St., Sackville, NB E4L 1G7, Canada; Gespe’gewa’gi Institute of Natural Understanding, 1 Marshall Way, Listuguj, QC, G0C 2R0, Canada; Department of Biology, Faculty of Forestry and Environmental Sciences, and Canadian Rivers Institute, University of New Brunswick, 28 Dineen Drive, Fredericton, NB, E3B 5A3, Canada; Department of Biology, Acadia University, 33 Westwood Avenue, Wolfville, NS, B4P 2R6, Canada; Department of Biology, Mount Allison University, 62 York St., Sackville, NB E4L 1G7, Canada

**Keywords:** Atlantic salmon, critical thermal maximum, growth rate, local adaptation, thermal acclimation, thermal performance, thermal variability

## Abstract

Temperature in many natural aquatic environments follows a diel cycle, but to date, we know little on how diel thermal cycles affect fish biology. The current study investigates the growth, development and physiological performance of wild Atlantic salmon collected from the Miramichi and Restigouche rivers (NB, Canada). Fish were collected as parr and acclimated to either 16–21 or 19–24°C diel thermal cycles throughout the parr and smolt life stages. Both Miramichi and Restigouche Atlantic salmon parr grew at similar rates during 16–21 or 19–24°C acclimations. However, as smolts, the growth rates of the Miramichi (−8% body mass day^−1^) and Restigouche (−38% body mass day^−1^) fish were significantly slower at 19–24°C, and were in fact negative, indicating loss of mass in this group. Acclimation to 19–24°C also increased Atlantic salmon *CT*_max_. Our findings suggest that both life stage and river origin impact Atlantic salmon growth and performance in the thermal range used herein. These findings provide evidence for local adaptation of Atlantic salmon, increased vulnerability to warming temperatures, and highlight the differential impacts of these ecologically relevant diel thermal cycles on the juvenile life stages in this species.

## Teaser

We show here that wild Atlantic salmon parr and smolt from thermally distinct river systems were impacted differently by cooler and warmer thermal variability. Growth, thermal tolerance and swimming performance were all impacted by river of origin, thermal acclimation temperature and life stage, highlighting the potential need for more river specific conservation guidelines.

## Introduction

Ectotherm physiology is strongly influenced by ambient temperature. Suboptimal temperatures can stress fishes, reduce their feed intake and consequently their growth (Jonsson *et al.,* 2001; Handeland *et al.,* 2008; Breau *et al.,* 2011; Volkoff and Rønnestad, 2020). Growth rate has been a particularly important area of research because overall size of the fish can impact wild fish population age-size structure, population dynamics and inter-species relations (Thackeray *et al.,* 2013; Carozza *et al.,* 2019; Huang *et al.,* 2021). The current understanding of temperature effects on growth rate in fishes is generally based on individuals acclimated to stable temperatures (Elliott and Hurley, 1997; Martell and Kieffer, 2007; Chadwick and McCormick, 2017; Scheuffele *et al.,* 2021). However, thermal stability is an unnatural condition in temperate rivers where temperature oscillates (3–15°C amplitude) diurnally (Lynch *et al.,* 1984; Malcolm *et al.,* 2004). Diel thermal cycles have been shown to influence food intake (Diana, 1984; Flodmark *et al.,* 2004), digestion efficiency (Mehner *et al.,* 2011; Coulter *et al.,* 2016) and routine metabolic rate (Oligny-Hébert *et al.,* 2015; Morash *et al.,* 2018), all factors that influence growth rate. In addition, exposure to cycling temperatures compared to stable temperature acclimation in the laboratory have been shown to alter aspects of physiology of Atlantic salmon such as their routine metabolic rate (Beauregard *et al.,* 2013; Morash *et al.,* 2018), fatty acid composition (Ge *et al.,* 2021) and haematology (Tunnah *et al.,* 2017) and have been reviewed elsewhere (Morash *et al.,* 2021). Despite the biological significance and ecological relevance of diel thermal cycles, compared to our understanding of the effects of stable temperatures, relatively little is currently known about their impacts on overall growth and development of fishes.

The Atlantic salmon is a keystone fish species that supports the culture, economy and ecology of Nordic countries and North America (Gardner Pinfold, 2011; Myrvold *et al.,* 2019). In the past 30 years, however, there have been significant declines in their global population and average body size, owing at least partly to climate warming (Swansburg *et al.,* 2002; COSEWIC, 2010; Mills *et al.,* 2013; Chaput *et al.,* 2019; DFO, 2019; Dadswell et al. 2021). In one of the most productive river systems for Atlantic salmon, the Miramichi River (NB, Canada), water temperature can reach and even exceed 30°C in July/August, with diel thermal cycles as wide as 9°C (Caissie *et al.,* 2013; Corey *et al.,* 2017). The typical summer diel thermal cycle here spans ~17–22°C with mean of summer maximums across 21 sites of 29.5°C (Caissie *et al.,* 2013). Laboratory experiments of Atlantic salmon parr (juvenile freshwater life stage) acclimated to constant temperatures have shown that water temperatures of 16–20°C elicit the fastest growth whereas temperatures between 22 and 27°C limit growth (Elliott and Hurley, 1997; Jonsson *et al.,* 2001). Therefore, water temperatures in the Miramichi River are often outside the thermal optima for wild Atlantic salmon growth. In another highly productive Atlantic salmon river, the Restigouche River (DFO, 2022), bordering northern New Brunswick and Quebec, overall water temperature is cooler (mean of summer maximums across 21 sites 25.4°C) than the Miramichi River and has a smaller variability throughout the day (Caissie *et al.,* 2013). To date, the effects of different thermal cycles on fish in ecologically, and importantly, thermally distinct streams are still unknown. However, recent *in situ* evidence from Atlantic salmon parr from rivers with different baseline thermal regimes shows variations in the temperature for the onset of behavioural thermoregulation (Corey *et al.,* 2020). These differences in behaviour in salmon parr from thermally and geographically distinct streams suggest that the thermal nature of their natal river may impact their tolerance to temperature stress that is likely underpinned by aspects of their physiology.

The effects of water temperature are perhaps most influential during the smolt life stage of Atlantic salmon (Gillis *et al.,* 2023). Smolts are juveniles preparing to transition from freshwater phenotype to saltwater phenotype during migration from freshwater streams to the ocean. The relationship between water temperature and initiation of smolt migration has been speculated to be related to a specific and/or cumulative temperature threshold but can also be impacted by day length and water discharge rates (McCormick *et al.,* 1998; Whalen *et al.,* 1999; Zydlewski *et al.,* 2005; Teichert *et al.,* 2020; Frechette *et al.,* 2022). Predictive models suggest that the mean temperature in the first 90 days of the year is a significant driver of the variation in onset of smolt migration timing across streams within a watershed (Frechette *et al.,* 2022). The survival success of the downstream migrants is also known to be dictated by environmental temperatures, and the smolts’ ability to meet certain ‘smoltification windows’ that are largely driven by temperature (McCormick *et al.,* 1997). While Atlantic salmon smolt migration generally occurs when water temperature remains < 15°C, cases of exposure to much warmer water temperatures are beginning to emerge. For example, in hydropower regulated rivers in the southern range of the species, it is not uncommon that the water temperature rises > 20°C towards the end of smolt migration period, especially for smolts for which efficient migration is delayed by downstream passage issues at hydropower dams (e.g. Babin *et al.,* 2020). Similar ‘high-temperature events’ during smolt migration have also recently been observed in unregulated natural rivers such as the Petitcodiac River in eastern Canada where water temperature rapidly exceeded 20°C during the peak of the smolt migration (personal communication, K. Samways, University of New Brunswick), or may occur when smolts are being trapped in downstream by-pass holding tanks with a smaller water volume while waiting for handling or transport downstream (MacLean, 2023). Exposure to rapidly changing and cycling temperatures is likely to become more common in the future due to warming climate scenarios (Borggaard *et al.,* 2019; Thorstad *et al.,* 2021; IPCC, 2022). Currently, in both the Atlantic salmon parr and smolt life stages, the effects of growth and metabolism under these conditions remain untested.

In this study, we investigated how wild Atlantic salmon parr and smolt growth and development differ at two ecologically relevant diel thermal cycles: 16–21 vs 19–24°C. We chose these thermal cycles as they reflect natural summer temperature conditions that are readily observed in both cool and relatively warmer rivers in Atlantic Canada (Caissie *et al.,* 2013), while simultaneously representing a possible transition from ‘optimal’ to ‘physiologically stressful’ conditions, respectively (see e.g. Elliott and Elliott, 2010; Breau *et al.,* 2011). We focused on comparing growth of salmon from the Miramichi River (a relatively ‘warmer’ river) to those from an on average ~4°C cooler, neighbouring Atlantic salmon-producing river system, the Restigouche River (Caissie *et al.,* 2013). We predicted that Restigouche salmon, compared to Miramichi salmon, would develop more poorly, and grow more slowly at 19–24°C (vs 16–21°C) because this warmer thermal cycle is currently beyond the temperatures they typically experience in their natal river, but could represent a future warmer river scenario. We also predicted that Miramichi salmon would develop and grow poorly at 19–24°C (vs 16–21°C) but to a much lesser extent compared to the Restigouche fish owing to their warmer natal environment. The assessment was done primarily to obtain a better understanding of between-population differences in the parr life stage. However, we continued the assessment using the same thermal comparisons through the smolt life stage, realizing that this thermal exposure is currently rare for wild smolts, but not unforeseen during natural smolt migration period especially considering future climate change scenarios. In addition, some of the southernmost populations of Atlantic salmon smolts in Maine, USA, are already migrating in these warmer temperatures (personal communication, G. Goulette, NOAA). We assessed salmon growth rate, Fulton’s condition factor, anaerobic performance and ability to tolerate acute warm exposures.

## Materials and Methods

### Animal collection, care and acclimation

We collected a total of 200 wild Atlantic salmon (age 1+ and 2+; parr that had spent two or three summers in the freshwater streams, respectively) from the Rocky Brook (*n* = 100; an ~500-m section of third-order stream of the Miramichi River at the collection site 46°43′2.34″N; 66°38′58.17″W) in October 2018 and the Chain of Rocks Brook (*n* = 100; an ~500-m section of a third-order stream of the Restigouche River at the collection site; 47°57′54.9″N; 67°11′43.0″W) in September 2019 by electrofishing over 2 days (Fisheries and Oceans Canada (DFO) permits SG-RHQ-18-158 and 20 190 812-009-01-S-P, respectively). We transported the salmon parr to the Crabtree Aqualab at Mount Allison University (NB, Canada) using a 750-L insulated transport tank filled with river water (~15°C), which was kept oxygenated by periodically injecting oxygen as required to maintain a minimum oxygen saturation of 9 mg/L. Upon arrival, we transferred the salmon parr into 300-L circular fibreglass tanks (60 cm tall, 92 cm diameter). We provided tanks with recirculating freshwater, at a 12:12 light/dark photoperiod. We initially fed salmon to satiation once daily with a diet of crushed freeze-dried krill to mimic more natural food as they do not directly take commercial fish pellets. We gradually transitioned their diet to pelleted feeds (EWOS Enviroclean pigmented pellets) by mixing it in with the freeze-dried krill at increasing concentrations. After 1 month of laboratory acclimation at 16–21°C, we tagged each salmon with a unique visible implant elastomer (Northwest Marine Technology) in their dorsal fin. Subsequently, we divided salmon into different tanks based on their river origin and designated acclimation temperatures (the 16–21 or 19–24°C diel thermal cycle). We simulated diel thermal cycles with a temperature change rate of ~0.42°C h^−1^, a temperature maximum at 19:00 h and minimum at ~07:00 h. Mid-daylight temperatures at 13:00 and mid-nighttime temperatures at 1:00 were 18.5°C and 21.5°C, respectively. We acclimated salmon to these thermal conditions for at least 1 month after tagging before conducting further experiments. All care and subsequent experimental procedures were approved by Mount Allison Animal Care Committee following guidelines from Canadian Council on Animal Care (protocol #101929).

### Experimental methods: growth, development and survivorship

Approximately once a month from 15 November 2018 to 25 September 2019 for Miramichi fish and 15 October 2019 to 23 April 2020 for Restigouche fish, we measured and recorded salmon mass, fork length and life stage, and calculated their growth rate (% body mass (BM) day^−1^) and Fulton’s condition factor (*k*; Eq. 1). Before each measurement, we fasted the salmon for 24 h then anesthetized them in a 5-L solution of 0.1 g L^−1^ tricaine mesylate buffered with 0.2 g L^−1^ sodium bicarbonate. Once anesthetized, weighed and measured, we screened individuals for the presence or absence of sexual precocity and classified their life stage (parr, transitioning to smolt or full smolt). Salmon with a distinct silvery body without parr marks were considered smolts (McCormick, 2012). Any fish mortalities were recorded daily throughout the experiment.

(1) \begin{equation*} K=\frac{Mass\left(\mathrm{g}\right)}{Fork\ length{\left(\mathrm{mm}\right)}^3}\cdot{10}^5 \end{equation*}

### Anaerobic capacity

We assessed anaerobic capacity by chasing fish (parr [*n* = 7–11] and smolts [*n* = 7–8] from each combination of temperature and river origin; see figures for exact *n* for each treatment) and recording the time taken until they exhaust (‘Time to Exhaustion’; Kelly *et al.,* 2014). Unique individuals were used for each trial and fish were not repeatedly measured. Individuals were chased in a bucket (42 cm high, 48 cm diameter) filled with continuously aerated water at 18.5 or 21.5°C for salmon acclimated to 16–21 or 19–24°C cycles, respectively. We chased salmon by pinching their tail periodically until they were exhausted (unresponsive and can be emersed for 5 s without struggling). After the chase test, salmon were allowed to rest for 3–5 min before being returned to their original acclimation tank.

### Acute thermal tolerance

We assessed acute thermal tolerance (*CT*_max_) (Desforges *et al.,* 2023) in parr (*n* = 6–11) and smolts (*n* = 8) sampled from each combination of acclimation temperature and river origin. Unique individuals were used for each trial and fish were not repeatedly measured. We first removed fish from their 16–21 or 19–24°C acclimation tank into 18.5 or 21.5°C (the mean of each thermal cycle) aerated water baths, respectively. The water bath was then warmed by 0.3°C per minute and *CT*_max_ was defined as the temperature where fish lost equilibrium (Corey *et al.,* 2017; Morgan *et al.,* 2018). Salmon were then transferred to a cool (16°C), aerated recovery bucket until they regained equilibrium before being returned to their original acclimation tank.

Due to a mechanical failure within our recirculation system, the Restigouche River smolts died prior to the completion of the time to exhaustion and *CT*_max_ tests. Therefore, no data are available for these two metrics for this group.

### Statistical analysis

We conducted all statistical analyses in R (2021; version 3.6.2).

### Growth rate

We first related salmon mass (response variable) to growth time (continuous predictor), acclimation temperature and river origin (categorical predictors) using linear mixed-effects model fitted with the function ‘lme’ (Pinheiro *et al.,* 2016). We then tested the significance (*α* = 0.05) of each predictor and their interaction on growth rate (model slope) using ANOVA (Type-III). We fit separate growth models for parr and smolts due to potentially different growth behaviour between life stages. For further insights into the effect of temperature, we compared growth rates of 16–21 and 19–24°C acclimated salmon from each origin and life stage combination, using pairwise *t*-tests. We accounted for the multiplicity of such tests by adjusting their significance or alpha level (from *α* = 0.05 into *α*_adj_) using the Benjamini–Hochberg (BH) procedure with a false discovery rate of 0.05.

### Survivorship

We compared survivorship of salmon from different origins and acclimation temperatures, by comparing (pairwise) their specific Kaplan–Meier survival curves using log-rank tests (Therneau, 2021). To account for the multiplicity of tests, we adjusted alpha levels based on the BH procedure described above.

### Condition factor (*k*)

We analysed how condition factor (response variable) depended on sampling day, acclimation temperature and their interactions (all categorical factors) using a non-parametric approach due to violations of assumptions, specifically using the ‘nparLD’ model (Noguchi *et al.,* 2012). We fit one ‘nparLD’ model for each origin that had different sampling days. At each time point, we additionally tested for the effect of temperature on condition factor of each origin group, using Mann–Whitney *U* tests. We adjusted alpha levels using the BH procedure, as above.

### Time to exhaustion and *CT*_max_

We first analysed how ‘Time to Exhaustion’ and *CT*_max_ (response variables) were affected by acclimation temperature (first factor), and the specific combination of salmon origin and life stage (second factor). This combination method allowed us to conduct our analysis without data from Restigouche smolts that were omitted to avoid temperature bias due to the lack of data from 16–21°C smolts. However, since temperature was found to be statistically insignificant for ‘Time to Exhaustion’, we omitted temperature as a factor in the ‘Time to Exhaustion’ model. The insignificance of temperature indicates that the available 19–24°C data for Restigouche smolts can be a fair representative for the smolts and Restigouche group. Using the 19–24°C data for Restigouche smolts, a subsequent analysis was conducted with only Life Stage and Origin as factors to ‘Time to Exhaustion’. We assessed significance (*α* = 0.05) of all factors to *CT*_max_ and time to exhaustion non-parametrically using permutation-based ANOVA (Type-III) with the function ‘aovp’ (Wheeler and Torchiano, 2016).

## Results

### Growth rate

**Figure 1:**
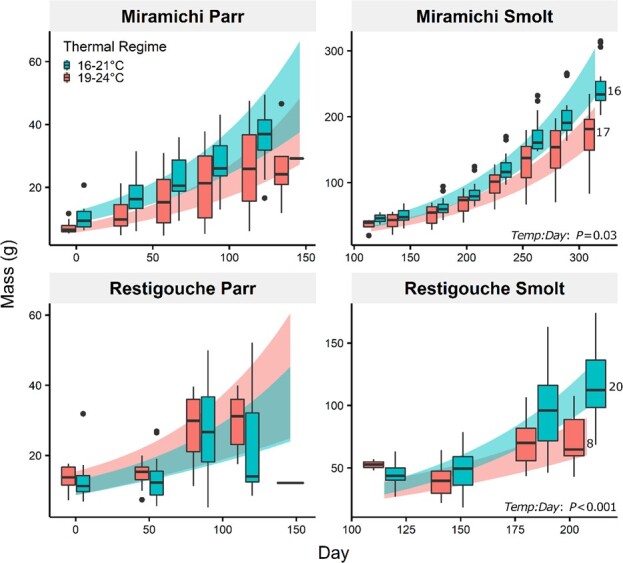
Growth of Atlantic salmon (*Salmo salar*) collected from the Miramichi River and Restigouche River acclimated to 16–21°C (turquoise) and 19–24°C (pink) diel thermal cycles, throughout the parr and smolt life stages. Day 0 represents the beginning of the experimental acclimation of the parr. Boxplots’ central horizontal line represents the median; upper and lower hinges represent first and third quartiles; whiskers represent maximum/minimum observation within 1.5-fold of the interquartile range. Coloured bands reflect 95% confidence intervals. *P* values result from pairwise comparisons of growth rates (slope curve) between temperature groups. Beside each boxplot at the last time point is the *n* for that group.

Salmon grew exponentially over time at rates that varied depending on acclimation temperature cycle for smolts (Time: Acclimation temp; *P* = 0.001) and river origin for parr (Time: Origin; *P* = 0.009; Supplementary Table S1; Fig. 1). Parr from the Miramichi River, on average, grew 32% faster, at 1.11% BM day^−1^ vs 0.81% BM day^−1^ for Restigouche parr (Table 1). Parr from both origins grew at rates that were not significantly different between acclimate temperature cycles (Time: Acclimation temp: Origin; *P* = 0.905), unlike smolts (Time: Acclimation temp: Origin; *P* = 0.005). Smolts from the Restigouche River at 19–24°C grew ~38% slower at 0.90% BM day^−1^ compared to 1.31% BM day^−1^ at 16–21°C (*P* < 0.001). In contrast, smolts from the Miramichi grew only marginally slower at 19–24 (−8%) than 16–21°C (*P* = 0.03).

### Survivorship

**Figure 2:**
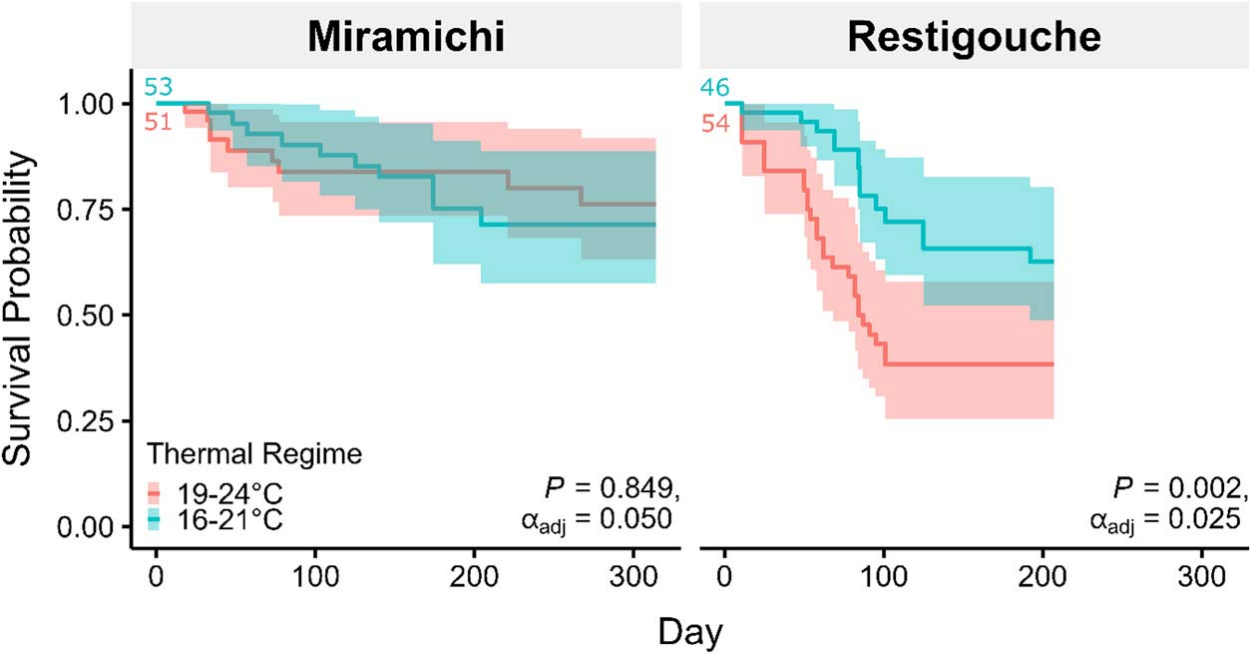
Survival probabilities of wild Atlantic salmon (*Salmo salar*) collected from the relatively warm Miramichi River and the cooler Restigouche acclimated to 16–21°C (turquoise) and 19–24°C (pink) diel thermal cycles. Coloured numbers represent the starting number of fish for each group. Coloured bands reflect 95% confidence intervals for survival probabilities. Crosses (+) indicate when certain individuals were withdrawn from the study for reasons unrelated to acclimation temperature cycle; such individuals do not affect survival probability. *P* values shown result from log-rank tests comparing survival curves at 16–21 and 19–24°C. The alpha level (*α* = 0.05) was adjusted (into *α*_adj_) using the Benjamini–Hochberg procedure to account for multiple pairwise comparisons.

Restigouche salmon parr and smolt life stages were less likely to survive at the warmer 19–24°C compared to 16–21°C (Fig. 2), with a statistically lower survivorship curve (*P* = 0.002; *α*_adj_ = 0.025; Supplementary Table S2). Restigouche salmon had a shorter average survival time at 19–24°C (57 days) than at 16–21°C (89 days), with fewer surviving until the end of the experiment (~40% vs ~60%, respectively; Supplementary Table S3). In contrast, Miramichi salmon had similar odds of surviving 19–24°C (76%) and 16–21°C (71%) until the end of the experiment. For Miramichi salmon, survival probability across the life cycle did not significantly differ between the temperature cycles (*P* = 0.849, *α*_adj_ = 0.050).

### Fulton’s condition factor (*k*)

**Figure 3:**
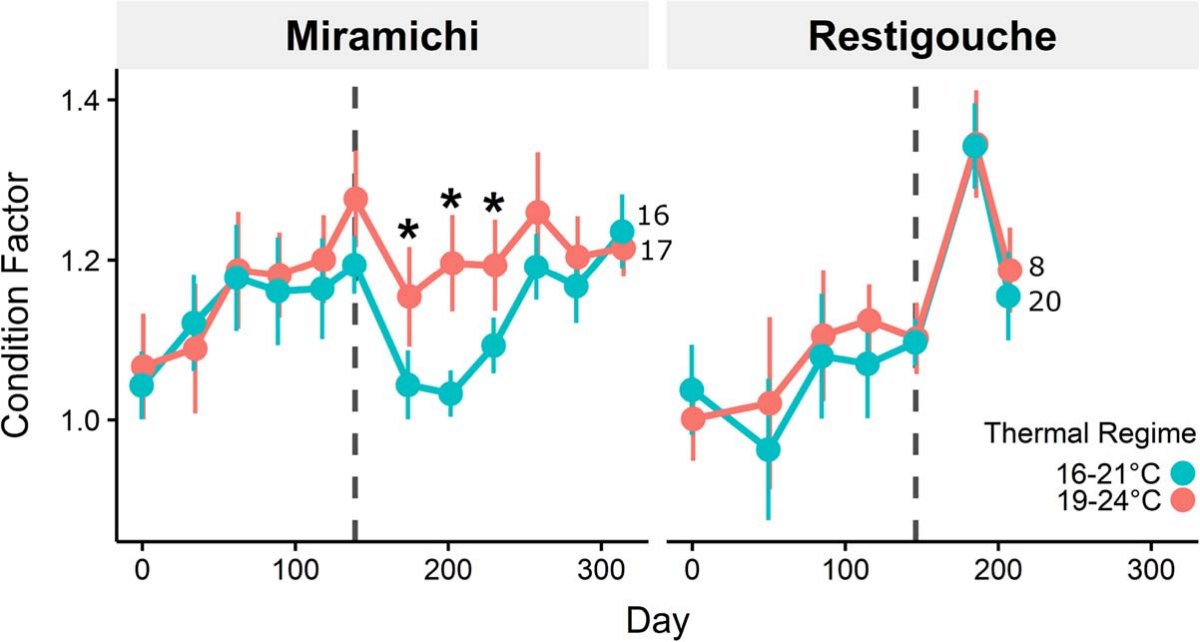
Condition factor (*k*) of Atlantic salmon (*Salmo salar*) collected from the Miramichi River and Restigouche River, acclimated to 16–21°C (turquoise) and 19–24°C (pink) diel thermal cycles throughout the parr and smolt life stages. Vertical dashed lines indicate the median date for parr–smolt transformation. Points represent means; error bars represent 95% confidence interval of means; asterisks mark significant differences in *k* between temperature groups assessed using Mann–Whitney *U* tests with alpha levels adjusted using the Benjamini–Hochberg procedure to account for multiple pairwise comparisons. Numbers of fish (*n*) for each treatment groups are shown beside their right-most point.

Regardless of river origin, salmon acclimated to 19–24 and 16–21°C generally had similar condition factors, except at specific time points for Miramichi fish (Acclimation temp: Time; *P* < 0.001; Supplementary Table S4). In each of the three months (days 174, 202 and 230, respectively) following smolting (median smolting Day 134), condition factor for Miramichi salmon was significantly higher in 19–24 than 16–21°C (*P* < *α*_adj_; Supplementary Table S5; Fig. 3). Condition factor then returned to similar values between temperature cycles, as observed for parr (Days ≤ 139). For Restigouche salmon, a similar temporal trend in condition factor was initially observed (Fig. 3). However, we did not observe a divergence in condition factor between temperature cycles after smolting occurred in Restigouche salmon, due to early termination of the project, and not necessarily the absence of such a phenomenon. We did not find significant differences between temperature cycles (Acclimation temp; *P* = 0.483 and Acclimation temp: Time; *P* = 0.229) in Restigouche salmon.

**Table 1:** Growth rates (% body mass (BM) day^−1^) for Atlantic salmon (*Salmo salar*) collected from the Miramichi River and Restigouche River, acclimated to 16–21 or 19–24°C diel thermal cycles

Life stage	Origin	Acclimation temperature (°C)	Growth rate (%BM day^−1^)	% Diff. in growth rates (16–21 vs 19–24°C)
Parr	Miramichi	16–21	1.07 ± 0.21	6.8
		19–24	1.15 ± 0.18	
	Restigouche	16–21	0.78 ± 0.23	6.0
		19–24	0.83 ± 0.31	
Smolt	Miramichi	16–21	0.99 ± 0.05	7.7*
		19–24	0.92 ± 0.05	
	Restigouche	16–21	1.31 ± 0.12	37.7*
		19–24	0.90 ± 0.20	

Growth rate values are presented alongside 95% confidence intervals. Asterisks (*) denote significant (pairwise *t*-test; *P* < 0.05) differences in growth rates.

### Anaerobic capacity

**Figure 4:**
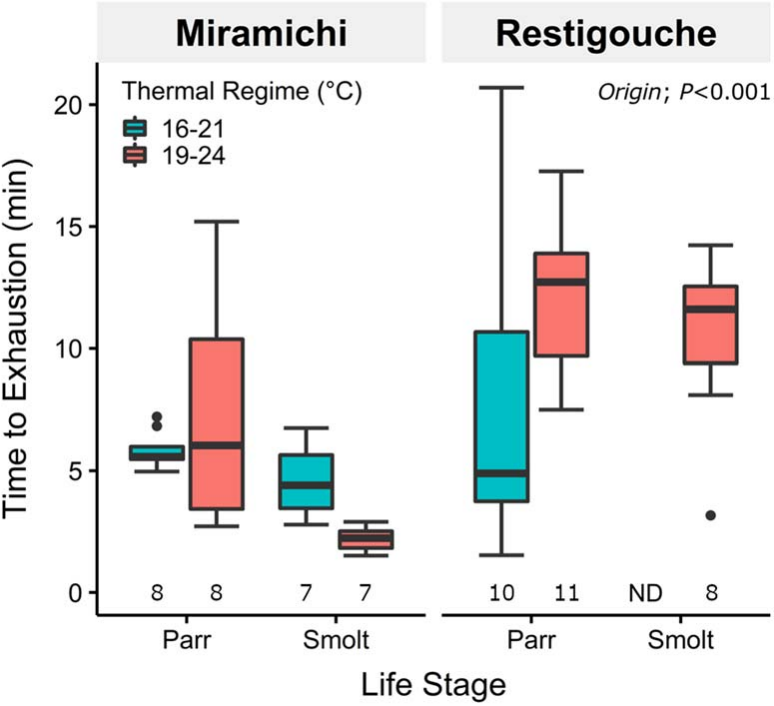
Time to exhaustion for chased Atlantic salmon (*Salmo salar*) collected from the Miramichi and Restigouche River, acclimated to 16–21°C (turquoise) and 19–24°C (pink) diel thermal cycles, throughout the parr and smolt life stages. Number of fish used for each group presented along the *x*-axis. Boxplots’ central horizontal line represents the median; upper and lower hinges represent first and third quartiles; whiskers represent the maximum/minimum observation within 1.5-fold of the interquartile range. ND denotes no data. *P* values shown are results from a permutation-based ANOVA (Type-III) model.

Time to exhaustion appeared independent of acclimation temperature cycle (Acclimation Temp: *P* = 0.345; Fig. 4) but dependent on salmon life stage and origin (Origin & Life Stage: *P* < 0.001; Fig. 4). Further analysis showed that origin was a major driver of the difference in time to exhaustion (Origin; *P* < 0.001). On average, Restigouche parr exhausted after ~13 min of chasing, significantly longer than the ~7-min average for Miramichi parr (permutation-based test; *P* = 0.027) and ~3-min average for Miramichi smolts (*P* < 0.001).

### Acute thermal tolerance

**Figure 5:**
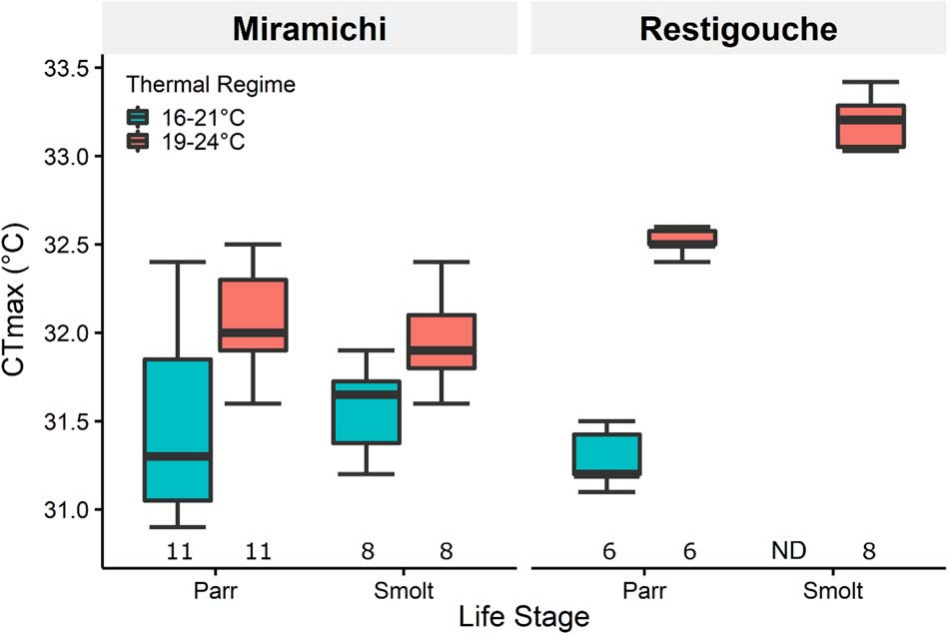
*CT*_max_ of Atlantic salmon (*Salmo salar*) collected from the Miramichi River and Restigouche River acclimated to 16–21°C (turquoise) and 19–24°C (pink) diel thermal cycles throughout the parr and smolt life stages. Number of fish used for each group presented on the *x*-axis. Statistical significance of salmon life stage and river origin as one factor (*P* = 0.238), acclimation temperature as another (*P* < 0.001), and their interactions (*P* = 0.006) were assessed using permutation-based ANOVA (Type-III); analysis excludes data from Restigouche smolts from either thermal cycle. Boxplots’ central horizontal line represents the median; upper and lower hinges represent first and third quartiles; whiskers represent the maximum/minimum observation within 1.5-fold of the interquartile range. ND denotes no data.

Acute critical thermal tolerance, as assessed using *CT*_max_ (Fig. 5), was unaffected by salmon river origin and life stage as a combined factor (Origin & Life Stage; *P* = 0.238; Supplementary Table S6), but significantly increased with warmer cycling acclimation temperatures (Acclimation temp; *P* < 0.001). On average, individuals at 19–24°C (excluding Restigouche smolts) had a *CT*_max_ ~1°C higher than those at 16–21°C. However, the increase in *CT*_max_ in the 19–24°C groups was significantly impacted by the interaction of origin & life stage (Acclimation temp: (Origin & Life Stage); *P* = 0.006). Average *CT*_max_ at 19–24 (vs 16–21°C) was higher by 1.2°C for Restigouche parr, while this number was only 0.6°C and 0.5°C for Miramichi parr and smolts, respectively.

## Discussion

Scientists’ understanding on the effects of temperature on fishes has been largely based on experiments conducted at stable temperatures, even though natural thermal environments have clear diel cycles. Here, we investigated the effects of two ecologically relevant diel thermal cycles (16–21 and 19–24°C) on growth and development of wild Atlantic salmon originating from two thermally distinct and socioeconomically important rivers in New Brunswick, Canada (Restigouche and Miramichi). We found that parr growth was similar between acclimation temperatures within each river, but Restigouche River parr grew significantly slower than Miramichi parr. In contrast, growth rate and survivorship for smolts decreased at 19–24 relative to 16–21°C in salmon from both rivers, but particularly for those from the cooler Restigouche River. Although the 19–24°C cycle appears to adversely affect salmon smolt growth, we did not observe any effect of thermal cycle on overall condition factor (*k*). Anaerobic performance was also not impacted by different thermal cycles, but Restigouche River salmon took longer to exhaust than those from the Miramichi. In contrast, *CT*_max_ overall was higher in salmon in the 19–24°C thermal cycle, but the differences were dependent on river of origin and life stage. Atlantic salmon parr from different river systems appear to respond differently to these relevant thermal cycles for several of the physiological metrics. The physiological impacts of these thermal cycles on fish from different river systems will provide valuable insight for predicted future warming.

There was no effect of our two natural thermal cycling acclimation conditions on the growth of salmon parr within each river of origin. However, parr from the Restigouche River grew 32% slower than those from the Miramichi on average. We initially expected an overall decrease in growth at 19–24°C based on past research using stable acclimation temperatures. For example, Norwegian Atlantic salmon parr acclimated to a range of stable temperatures grew fastest at 16 to 20°C, above which growth rate declined until it eventually ceased at 24 to 27°C (Jonsson *et al.,* 2001). In another study on European Atlantic salmon parr, growth rate peaked at a stable 16°C, then decreased to zero at ~ 23°C (Elliott and Hurley, 1997). However, the optimal temperature for growth in juvenile salmon, determined under controlled conditions, is lower, ranging from 15 to 20°C (Elliott and Hurley, 1997; Jonsson *et al.,* 2001; Elliott and Elliott, 2010). In another study, the thermal optimum (*T*_opt_) for Atlantic salmon parr was determined to be 15–19°C (Gillis *et al.,* 2023). These differences between our study and others could possibly be due to feed availability and consumption. Laboratory experiments may also underestimate the effects of climate change on *T*_opt_ when compared to field-based measurements where things like food availability are not controlled (Childress and Letcher, 2017). Here, we fed the fish *ad libitum* each day so food would not be a constraining factor. In addition, our warmest thermal cycle was within the upper thermal limit for juvenile salmon feeding in the laboratory or the wild (22–24°C; Cunjak *et al.,* 1993; Breau *et al.,* 2011). Smaller ration size can decrease the *T*_opt_ for growth in salmonids (Brett *et al.,* 1969; Elliott, 1976); therefore, our high rate of feeding may have increased the *T*_opt_ for growth in the laboratory.

It is also possible that the salmon in this study possessed a relatively broad *T*_opt_ range following acclimation to our variable thermal environments, and this may help explain the similar growth rates of parr from a given river of origin at 16–21 and 19–24°C. Diel thermal cycles tend to broaden the optimum thermal range for maximum performance as shown in green sturgeon (*Acipenser medirostris*; Rodgers *et al.,* 2018) and alpine newt larvae (*Triturus alpetris*; Měráková and Gvoždík, 2009). However, cycles also reduce maximum performance in fruit flies (*Drosophila melanogaster*; Kjaersgaard *et al.,* 2012) and a frog species (*Bombina orientalis*; Arrighi *et al.,* 2013). It is possible that animals in diel thermal cycling environments benefit from a wider thermal performance curve (da Silva *et al.,* 2019), adopting a more thermal generalist strategy vs thermal specialist (Rodgers *et al.,* 2018). The thermal generalist strategy may be used by our salmon parr, explaining their similar growth rate at 16–21 and 19–24°C. Alternatively, if the *T*_opt_ lies in the overlapping range of our thermal cycles (19–21°C), then both groups would have been exposed to their *T*_opt_ for similar amounts of time each day enabling them to grow at similar rates.

Local adaptation to distinct thermal environments may also explain why our Atlantic salmon parr did not exhibit a decreased growth rate at 19–24°C as predicted by previous studies of Atlantic salmon from European origins (Elliott and Hurley, 1997; Jonsson *et al.,* 2001). Different habitats are expected to exert different selection pressures leading to different genotypic adaptations in salmonids (Dionne *et al.,* 2009; Weitemier *et al.,* 2021; Zillig *et al.,* 2021). The extent of local adaptation for salmon populations has been extensively discussed (in Garcia de Leaniz *et al.,* 2007; Fraser *et al.,* 2011; Primmer, 2011), and evidence for its occurrence has surfaced based on comparisons of physiology or genomics of adult (Taylor, 1991; Lee *et al.,* 2003; Unwin *et al.,* 2003) and juvenile (Zillig *et al.,* 2023) salmon from different locations. For example, Sockeye salmon (*Oncorhynchus nerka*) from warmer environments tend to have better swim performance at warmer temperatures (Eliason *et al.,* 2011). Atlantic salmon from warmer southern rivers possessed hearts more tolerant to acute warming (Gradil *et al.,* 2016, North American salmon) and cardiac capacity or maximum heart rate was also increased after warm acclimation (Anttila *et al.,* 2014, European salmon). The Atlantic salmon used in this study were a population from the warmer Miramichi River (summer *T*_mean_ 16–19°C; Caissie *et al.,* 2013) compared to English and Norwegian rivers (summer *T*_mean_ 9–16°C; Elliott and Hurley, 1997; Jonsson *et al.,* 2001) where most previous growth rate studies were conducted. We hypothesize that Miramichi River salmon may have developed the capability to continue to grow during the warmer summer conditions (19–24°C) compared to the Restigouche salmon. However, it is unclear whether such capability arises due to changes in genetics (local adaptation) or phenotypic plasticity (acclimation capacity) that arise from differences in developmental temperature (Zillig *et al.,* 2021; Andrews, 2008; Jonsson and Jonsson, 2011). Historically, evidence for local thermal adaptation for growth rate of Atlantic salmon, based on individuals incubated at similar temperatures then acclimated to different stable temperatures, is lacking (Jonsson *et al.,* 2001). The unimpaired growth of our salmon parr in laboratory conditions at 19–24 (vs 16–21°C) suggests that local thermal adaptation took place. Further study is needed to understand the facilitating mechanism and the variability of the capacity for Atlantic salmon to adapt to local watershed conditions and will be important for developing population-specific management practices (Gayeski *et al.,* 2018; Zillig *et al.,* 2021). This is especially important in systems such as the Miramichi where a previous long history of supplementation (i.e. stocking) may have influenced the once natural patterns of local adaptation (Wellband *et al.,* 2019). It is also important to keep in mind that the availability and development/emergence rates of prey, mainly different macroinvertebrates, also respond to water temperature, so the effects of different temperature regimes on parr growth may manifest differently *in situ* vs in a laboratory study.

In a more local context, we also found that salmon parr from the cooler Restigouche River overall grew significantly slower than those from the Miramichi. Growth of Restigouche smolts decreased significantly at 19–24°C compared to smolts from the Miramichi. Restigouche salmon, which are genetically distinct from salmon populations in other river systems across Atlantic Canada (Dionne *et al.,* 2009), inhabit a river system that is generally ~4°C cooler than the Miramichi (Caissie *et al.,* 2013). Their cooler habitat potentially reduces the need for an ability to grow optimally at warm temperatures, explaining the lower growth rate in parr compared to the Miramichi salmon and the reduction in their growth and survival rate in smolts at 19–24°C. Notably, we only observed the warm-associated decrease in growth rate in the smolt life stage. From a physiological perspective, older and larger fishes are generally believed to have reduced thermal tolerance (Cuenco *et al.,* 1985; Bjornsson *et al.,* 2001; Morita *et al.,* 2010; Audzijonyte *et al.,* 2020; Turko *et al.,* 2020; McKenzie *et al.,* 2021). From an ecological perspective, smolts leave the Restigouche River from the middle of May to late June at 6–16°C (Peppar, 1982), so it is unlikely that they would typically experience these temperatures in the wild at present. Regardless, our findings revealed the greater vulnerability of Restigouche salmon to warmer and relevant thermal cycles, which will be important to consider for the predicted warming climate. Indeed, Atlantic salmon in the Northeast United States Shelf have been assessed to have extremely high biological sensitivity and climate exposure making them vulnerable to changes in population productivity (Hare *et al.,* 2016). Our data will contribute to a knowledge base on the effects of climate warming on Atlantic salmon, which has been identified as an area of uncertainty in climate change predictions for this species (Borggaard *et al.,* 2019; Kocik *et al.,* 2022; Henderson *et al.,* 2023).

Despite differences in growth, overall condition factor throughout the growth periods remained unaffected by acclimation to either thermal cycle or between rivers. Our Atlantic salmon kept high Fulton’s condition (>1) even at the warm 19–24°C across both parr and smolt developmental stages. However, we did note that Miramichi salmon at 19–24°C maintained a higher Fulton’s condition in the few months after parr–smolt transformation compared to those at 16–21°C. Fulton’s condition factor has been a popular indicator of proximate body composition and overall fish condition (Baxter, 1998; Jin *et al.,* 2015; Naeem *et al.,* 2016). Typically, it decreases in fish in unfavourable environments (Árnason *et al.,* 2009; Mazumder *et al.,* 2016; Hvas *et al.,* 2017; Castaldo *et al.,* 2021). A general decline in Fulton’s condition during the post-smolt period may be attributed to the parr–smolt transformation as it has also been observed previously after smoltification (Shrimpton *et al.,* 2000; Stefansson *et al.,* 2008; Codabaccus *et al.,* 2011). The extent of decline in Fulton’s condition during this period, differing between anadromous vs landlocked populations (McCormick *et al.,* 2019), has been associated with differences in gill Na^+^/K^+^-ATPase (NKA) activity and NKAα1b essential for successful sea-entry of salmon (Björnsson *et al.,* 1989; McCormick *et al.,* 2019; Nisembaum *et al.,* 2020), and not necessarily their overall condition. Based on these studies, it is difficult to attribute differences in Fulton’s condition observed in the few months post-smolt as an indication of poorer condition at 19–24°C; such differences may instead suggest a temperature-sensitive smolting process for Atlantic salmon (Handeland *et al.,* 2000; Imsland *et al.,* 2014). It is also important to note that these fish remained in freshwater during this period. Fish with higher Fulton’s condition tend to possess greater acute thermal and hypoxia tolerance, and energy reserves (Mozsár *et al.,* 2015; Gallant *et al.,* 2017; Rees and Matute, 2018; Turko *et al.,* 2020) that may lead to increased physiological performance, but this was not tested here.

Despite differences in growth and some changes to Fulton’s condition throughout development, we did not observe any differences in anaerobic performance at the warm acclimation temperature cycle. The effects of different stable acclimation temperatures on fish anaerobic performance appear to be variable/inconsistent (Kieffer, 2000). The fuel for anaerobic activity, white muscle glycogen, generally remains at similar levels regardless of stable acclimation temperature (Kieffer *et al.,* 1994; Kieffer and Tufts, 1998; Day and Butler, 2005), although there are exceptions (Wilkie *et al.,* 1996). Warming increased burst swimming speed in striped mullet (*Muglis cephalus*; Rulifson, 1977) and goldfish (*Carassius auratus*; Johnson and Bennett, 1995), but not consistently in threespine sticklebacks (*Gasterosteus aculeatus*; Guderley *et al.,* 2001), and there was no effect on Atlantic salmon parr (Zathey, 2018). Furthermore, the time to exhaustion was not affected by acclimation temperature for four lake trout populations (*Salvelinus namaycush;*Kelly *et al.,* 2014). Notably, these studies used fish acclimated to stable temperatures, which may be different physiologically compared to those acclimated to ecologically relevant diel thermal cycles (Oligny-Hébert *et al.,* 2015; Morash *et al.,* 2018; Rodgers *et al.,* 2018). We showed that 16–21 and 19–24°C diel thermal cycles did not affect the time to exhaustion of Atlantic salmon, indicating unchanged anaerobic performance that could be important in overcoming velocity barriers, prey capture, and escaping predators. However, despite no effect of thermal acclimation on anaerobic performance, there would likely be differences in their aerobic capacity due to the thermal influences on aerobic metabolism (Pörtner and Knust, 2007). A warmer thermal acclimation profile may increase metabolic demands that might not be met by their capacity for oxygen uptake, which may force them to rely on anaerobic processes/substrates sooner than at cooler acclimation temperatures. Where anaerobic substrates are limiting, this could potentially limit repeat bouts of anaerobic activity at warmer temperatures.

Although time to exhaustion did not vary with acclimation temperature, it is overall higher in Restigouche salmon parr compared to those from the Miramichi. Variations in anaerobic capacity between fish populations are thought to be linked with differences in natal environment hydrology and predatory intensity (O’Steen *et al.,* 2002; Langerhans and DeWitt, 2004) and not necessarily to temperature. For example, mosquitofish (*Gambusia affinis*) in environments with larger predators have been found to have faster burst swim speed (Langerhans *et al.,* 2004). Similarly, Coho salmon (*Oncorhynchus kisutch*) in coastal areas, presumed to be more predator dense, have a higher burst swim speed compared to those in interior zones (Taylor and McPhail, 1985). In addition, domesticated rainbow trout (*Oncorhynchus mykiss*) have reduced burst swimming performance compared to their wild counterparts (Bellinger *et al.,* 2014). The higher anaerobic performance of Restigouche salmon may be the result of differences in their natal environment that is not temperature related. This possibility awaits further investigation.

Acute thermal tolerance (as measured through *CT*_max_) increased with warmer acclimation temperature as is typically observed in several species (Zhang and Kieffer, 2014; Comte and Olden 2016; Corey *et al.,* 2017). The *CT*_max_ of Restigouche and Miramichi salmon increased by ~0.5 and 1.2°C, respectively, for the +3°C warmer acclimation cycle, which is about the typical expected increase in *CT*_max_ for every 3°C found in other fishes (0.9–1.2; Beitinger *et al.,* 2000; Comte and Olden 2016; Morley *et al.,* 2019). For some fishes under warm temperatures, *CT*_max_ increased only slightly with warm acclimation (Morrison *et al.,* 2020; Reid and Riccairdi 2021), which could indicate that the fish is approaching its thermal ceiling and is unable to adjust *CT*_max_. Our data indicate that our temperature cycles were within the thermal breadth of performance for Atlantic salmon in both tested populations.

In conclusion, we found that the effects of these two ecologically relevant thermal cycles on growth and physiological performance were impacted by both river of origin and life stage of the salmon. Salmon parr within each river system appear to be able to grow just as well when exposed to chronically cooler or warmer thermal cycles, which contradicts the predicted decline in growth rate from research using stable temperature acclimations. However, overall growth of salmon from the cooler Restigouche River was significantly slower despite common conditions. These differences could be suggestive of local adaptation to distinct thermal regimes between these salmon populations. In addition, smolts from the Restigouche River appear to be more vulnerable to warmer thermal cycles than those in the Miramichi. The temperatures used in this experiment are currently unlikely to be experienced by smolts in either river; however, future changes in climate and/or anthropogenic changes to river structures that inhibit or slow smolt downstream passage could make this a reality in the future. Together, our findings should inform evidence-based decision-making in support of a river-by-river conservation approach for Atlantic salmon in the Gulf region. It is unknown if local thermal adaptation occurs across all/most Canadian Atlantic salmon populations, and this could be a rewarding area for future research.

## Supplementary Material

Web_Material_coae007

## Data Availability

Data generated or analysed during this study will be available in the Borealis Data Repository upon acceptance. https://doi.org/10.5683/SP3/3P5MAY
