# Supplementary material for: The impacts of diel thermal variability on growth, development and performance of wild Atlantic salmon (Salmo salar) from two thermally distinct rivers
Source: Conserv Physiol. 2024 Feb 12;12(1):coae007. doi: 10.1093/conphys/coae007 (PMC10939361; doi:10.1093/conphys/coae007)
Supplement: Web_Material_coae007 [file web_material_coae007.zip › Supplementary_Materials clean.pdf]

Supplementary Materials

Table S1: Mixed models for the growth of Atlantic salmon (*Salmo salar*).

Model-Life Stage	Parameters	SD/ Estimate*	PD (%)†	df_{num}	df_{den}	F	P
Lme-Parr	Random Effects						
	Intercept by Fish.ID	0.33					
	Time by Fish.ID	0.0030					
	Fixed Effects						
	Intercept-modifying parameters						
	Intercept	2.27 ± 0.11					
	Origin (Restigouche)	-0.14 ± 0.11	12.2 ± 9.6	1	53	6.47	0.014
	Acclimation temp (19-24ºC)	0.08 ± 0.11	7.4 ± 9.6	1	53	2.40	0.128
	Acclimation temp: Origin	-0.13 ± 0.11		1	53	5.75	0.020
	Slope-modifying parameters						
	Time (Days)	0.0096 ± 0.0012		1	187	267	<0.001
	Time: Origin	0.0015 ± 0.0012	32 ± 24	1	187	6.97	0.009
	Time: Acclimation temp	0.0003 ± 0.0012	6 ± 24	1	187	0.27	0.599
	Time: Acclimation temp: Origin	0.0001 ± 0.0012		1	187	0.01	0.905
	N: 57						
	n: 248						
Lme-Smolt	Random Effects						
	Intercept by Fish.ID	0.26					
	Time by Fish.ID	0.0002					
	Fixed Effects						
	Intercept-modifying parameters						
	Intercept	2.31 ± 0.13					

Origin	0.10 ± 0.13	9 ± 11	1	53	2.36	0.130
Acclimation temp	0.08 ± 0.13	7 ± 11	1	53	1.45	0.235
Acclimation temp: Origin	-0.14 ± 0.13		1	53	4.40	0.041
Slope-modifying parameters						
Time	0.0103 ± 0.0006		1	250	1172	<0.001
Time: Origin	-0.0008 ± 0.0006	15 ± 12	1	250	6.54	0.011
Time: Acclimation temp	-0.0012 ± 0.0006	23 ± 12	1	250	16.2	0.001
Time: Acclimation temp: Origin	0.0008 ± 0.0006		1	250	7.89	0.005
N: 57						
n: 311						

Values to the right of plus-minus signs (±) are 95% confidence intervals.

* Standard deviation (SD) and estimates are provided for random- and fixed- effect parameters, respectively. The fixed effect estimates describe the deviation of each factor level (e.g. 16-21 and 19-24°C) from their grand mean. The estimate's positive/negative sign apply to the reference factor level contained in brackets beside parameter names. Statistical significance of the fixed effect parameters were assessed using Type III ANOVA; $P \leq 0.05$ are bolded and considered statistically significant.

† PD describes the absolute percent difference in "slope" or "intercept" between factor levels.

11

Table S2: *P*-values (with α_{adj} subscripts) from log-rank tests comparing Kaplan Meier survival curves of Miramichi and Restigouche Atlantic salmon (*Salmo salar*) acclimated to 16-21 and 19-24°C diel thermal cycles.

Origin, Acclimation temperature	Miramichi, 19-24°C	Miramichi, 16-21°C	Restigouche, 19-24°C	Restigouche, 16-21°C
Miramichi, 19-24°C	-			
Miramichi, 16-21°C	$0.849_{0.050}$	-		
Restigouche, 19-24°C	$<0.001_{0.017}$	$<0.001_{0.008}$	-	
Restigouche, 16-21°C	$0.103_{0.033}$	$0.240_{0.042}$	$0.002_{0.025}$	-

P values less than the adjusted alpha level (α_{adj}) are bolded and considered statistically significant. The alpha level was adjusted using the Benjamini-Hochberg procedure to control for the false discovery rate (set to 0.05) of multiple hypothesis tests belonging to the same "family".

Table S3: Survival statistics for Atlantic salmon (*Salmo salar*) collected from Miramichi River and Restigouche River, acclimated to 16-21 and 19-24°C diel thermal cycles.

	Miramichi		Restigouche	
	16-21°C	19-24°C	16-21°C	19-24°C
Number of Fish				
Initial	53	51	46	44
Dead	10	9	15	26
Censored* [Survivors]	16	17	20	8
SP_{END}†	0.71	0.76	0.63	0.38
Mean Survival Time (Days)	114	88	89	57

* Censored fish are those whose death are unrelated to acclimation temperature (e.g. sacrificed for hematology, jumped out of tank).
† Likelihood of surviving until the end of the experiment.

Table S4: *P*-value of acclimation temperature, time, and their interaction as factors to condition factor of Atlantic salmon (*Salmo salar*) collected from the Miramichi River and Restigouche River, acclimated to 16-21 and 19-24°C diel thermal cycles.

Model-Origin	Factor	Wald-Type Statistic	df	P
nparLD-Miramichi	Time	253	11	<0.001
	Acclimation temp	6.28	1	0.012
	Acclimation temp: Time	42.5	11	<0.001
nparLD-Restigouche	Time	285	6	<0.001
	Acclimation temp	0.49	1	0.483
	Acclimation temp: Time	8.13	6	0.229

$P \leq 0.05$ are bolded and considered statistically significant.

Table S5: Condition factor (CF) and its corresponding Relative Treatment Effect (RTE) between Atlantic salmon (*Salmo salar*) collected from the Miramichi River and Restigouche River, acclimated to 16-21 and 19-24°C diel thermal cycle.

Origin	Day	19-24°C		16-21°C		Differences Between Thermal Regimes		
		CF	RTE	CF	RTE	ΔCF*	P†	α_{adj}§
Miramichi	0	1.07 ± 0.07	0.27	1.04 ± 0.04	0.20	0.02 ± 0.08	0.642	0.046
	34	1.09 ± 0.08	0.36	1.12 ± 0.06	0.41	0.03 ± 0.10	0.515	0.042
	62	1.19 ± 0.07	0.58	1.18 ± 0.07	0.57	0.01 ± 0.10	0.867	0.050
	89	1.18 ± 0.05	0.58	1.16 ± 0.07	0.53	0.02 ± 0.09	0.491	0.033
	118	1.20 ± 0.06	0.61	1.16 ± 0.06	0.54	0.04 ± 0.08	0.239	0.025
	139	1.28 ± 0.06	0.75	1.19 ± 0.04	0.59	0.08 ± 0.07	0.056	0.021
	174	1.15 ± 0.06	0.47	1.04 ± 0.04	0.20	0.11 ± 0.08	0.006	0.008
	202	1.20 ± 0.06	0.58	1.03 ± 0.03	0.17	0.16 ± 0.07	0.000	0.004
	230	1.19 ± 0.06	0.58	1.09 ± 0.03	0.30	0.10 ± 0.07	0.008	0.013
	258	1.26 ± 0.08	0.74	1.19 ± 0.04	0.58	0.07 ± 0.09	0.043	0.017
	284	1.20 ± 0.05	0.59	1.17 ± 0.05	0.51	0.04 ± 0.07	0.491	0.033
	314	1.21 ± 0.04	0.63	1.24 ± 0.05	0.69	0.02 ± 0.06	0.318	0.029
Restigouche	0	1.00 ± 0.05	0.22	1.04 ± 0.06	0.30	0.04 ± 0.08	0.628	0.029
	50	1.02 ± 0.11	0.34	0.96 ± 0.09	0.23	0.06 ± 0.14	0.440	0.014
	85	1.11 ± 0.08	0.50	1.08 ± 0.08	0.48	0.03 ± 0.11	0.754	0.036
	115	1.12 ± 0.05	0.55	1.07 ± 0.07	0.45	0.05 ± 0.08	0.367	0.007
	146	1.10 ± 0.04	0.47	1.10 ± 0.03	0.47	0.00 ± 0.05	0.977	0.043
	185	1.34 ± 0.07	0.92	1.34 ± 0.05	0.90	0.00 ± 0.09	0.977	0.050
	207	1.19 ± 0.05	0.70	1.15 ± 0.06	0.58	0.03 ± 0.08	0.511	0.021

CF means are presented alongside 95% confidence intervals (to the right of plus-minus signs "±"). For each CF mean, an RTE value is provided. RTEs, obtained from nparLD models, represent the probability for a CF of an observation in that group (e.g. Miramichi, 19-24°C at Day 0) to be greater than CF of an observation chosen at random from all fitted into the nparLD.

* ΔCF are presented in absolute values.

† *P* values are obtained from Mann-Whitney U tests that compares CF between acclimation groups at each time-point. P values less than the adjusted alpha level (α_{adj}) are bolded and considered statistically significant.

§ The alpha level was adjusted (α_{adj}) using the Benjamini-Hochberg procedure to control for the false discovery rate (set to 0.05) of multiple hypothesis tests belonging to the same "family".

Table S6: *P*-value for river origin & life stage as one factor, as well as rearing temperature as one factor, and their interactions, on the CT_{max} of Atlantic salmon (*Salmo salar*) collected from the Restigouche and Miramichi acclimated to 16-21 and 19-24°C diel thermal cycles.

Factor	df_{num}	df_{den}	F	P
(Origin & Life Stage)	2	44	1.49	0.238
Acclimation temp	1	44	41.9	<0.001
Acclimation temp: (Origin & Life Stage)	2	44	5.73	0.006

Statistical significance was assessed using permutation-based ANOVA (Type III). $P \leq 0.05$ are bolded and considered statistically significant.
